# Assessment of gold nanoparticles on human peripheral blood cells by metabolic profiling with ^1^H-NMR spectroscopy, a novel translational approach on a patient-specific basis

**DOI:** 10.1371/journal.pone.0182985

**Published:** 2017-08-09

**Authors:** Martina Palomino-Schätzlein, Hermenegildo García, Patricia Gutiérrez-Carcedo, Antonio Pineda-Lucena, José Raul Herance

**Affiliations:** 1 Laboratorio de Bioquímica Estructural, Centro de Investigación Príncipe Felipe, Valencia, Spain; 2 Instituto Universitario de Tecnología Química CSIC-UPV, Valencia, Spain; 3 Grup de Recerca en Imatge Mèdica Molecular, Vall d’Hebron Research Institute, CIBBIM-Nanomedicine, Departament de Medicina, Universitat Autònoma de Barcelona, Barcelona, Spain; 4 Unidad de Descubrimiento de Fármacos, Instituto de Investigación Sanitaria La Fe, Hospital Universitario i Politécnico La Fe, Valencia, Spain; Advanced Centre for Treatment Research and Education in Cancer, INDIA

## Abstract

Human peripheral blood cells are relevant ex vivo models for characterizing diseases and evaluating the pharmacological effects of therapeutic interventions, as they provide a close reflection of an individual pathophysiological state. In this work, a new approach to evaluate the impact of nanoparticles on the three main fractions of human peripheral blood cells by nuclear magnetic resonance spectroscopy is shown. Thus, a comprehensive protocol has been set-up including the separation of blood cells, their *in vitro* treatment with nanoparticles and the extraction and characterization of metabolites by nuclear magnetic resonance. This method was applied to assess the effect of gold nanoparticles, either coated with chitosan or supported on ceria, on peripheral blood cells from healthy individuals. A clear antioxidant effect was observed for chitosan-coated gold nanoparticles by a significant increase in reduced glutathione, that was much less pronounced for gold-cerium nanoparticles. In addition, the analysis revealed significant alterations of several other pathways, which were stronger for gold-cerium nanoparticles. These results are in accordance with the toxicological data previously reported for these materials, confirming the value of the current methodology.

## Introduction

Blood cells are interesting *ex vivo* models to study the pathophysiological state of diseases, and to predict the beneficial or toxic effect promoted by new therapies, with a high translationality to clinical studies by considering the patient’s specific characteristics. The reason is the fact that blood cells are altered in disease and can reflect the condition and state of different organs and tissues [1]. Thus, the study of the interaction of blood cells with medicines can provide us with an early indication of the effect of a certain therapy on the human body. For instance, erythrocytes or red blood cells (RBCs) have been used as disease models for assessing drugs as they are sensitive to many disorders, including diabetes, Wilson’s disease and Alzheimer’s disease [2–5]. Moreover, neutrophils have been analysed to obtain information about the diagnosis, mechanism of action and therapy of different diseases, such as tuberculosis, malaria, allergic reactions or tumours [6–12]. Furthermore, lymphocytes have shown to be altered in lung diseases, inflammatory processes leading to allergic diseases, during atherosclerotic plaque development, in cardiovascular diseases and during tumour progression [13–20].

Metabolomic profiling is a comprehensive method that allows the quantification of a large number of different metabolites in a single analysis in a non-targeted way that can provide useful information to study disease and the effect of treatments.[21–24] Proton nuclear magnetic resonance (^1^H-NMR) spectroscopy has proven fast and reproducible for obtaining good quality structural and semi-quantitative information about the metabolome of cells [21,25]. Metabolic profiling of cells has been previously applied to a wide range of *in vitro* models to help gain insight into basic and disease metabolisms, especially in combination with genomics and/or proteomics data [26]. Although some studies about the metabolic profile of blood cells can be found, to our knowledge, very limited data about blood cell analysis by NMR spectroscopy from patients available [27–35]. The analysis of the metabolic profile of blood cells could not only provide a method for identifying new biomarkers for disease diagnosis, but also for in vivo evaluating the effects of new therapeutic treatments (e.g., nanomedicines) at a patient level [22–24].

Nanomedicine is the application of nanotechnological systems to medicine. The impact of this technology has augmented dramatically over the last few years due to its applications (drug delivery, prevention of drug metabolisation, diagnostic agent, etc.) [36,37]. Thanks to its advantages, to date several nanometric systems have been approved for human use, and more than 240 are in different clinical trial phases. This situation creates the need to implement a wider range of methodological tools to optimize the design of new nanomaterials in early stages of their development and to assess their effect during clinical trials [38]. Blood is one of the first environments that comes into contact with a nanomedicine when it is injected or when it enters the bloodstream via other administration types, which makes the study of the interaction of nanoparticles with different blood components highly relevant. Comprehensive studies have been reported on the effect of nanomaterials on both the immune and coagulating systems. They include the analysis of the impact of these compounds on the morphology, cell cycle and proliferation of different types of blood cells [39–44]. Indeed, new nanomaterials are designed to make this interaction as controlled and advantageous as possible, and blood cells have even been employed as carrier cells for nanoparticles to reach their destiny more efficiently [45,46]. In this context, the focus of our study was to evaluate the potential of metabolomics by NMR to characterize the metabolic profile of peripheral blood cells before and after treatment with nanoparticles. To test our approach, we have chosen gold nanoparticles as model systems, because they are one of the most promising nanomedicines, that have been suggested for a wide range of different applications; e.g., medical imaging and therapies in cancer, neurodegenerative diseases or diabetes [47–58]. One of its most promising properties is its capacity to eliminate an excess of oxidant species generated in stress situations (antioxidant behavior), which is beneficial for many biomedical applications. Most of these applications involve a direct contact with peripheral blood, whose impact can be evaluated by our method. Several approaches have been proposed to maintain the structure of gold as nanoparticles and to prevent agglomeration by adjusting their properties for biomedical applications. For instance, to increase their biocompatibility and activity against oxidative stress, gold nanoparticles have been supported on ceria nanoparticles or assembled in chitosan [59–63]. A wide range of different medical applications, including glucose sensors, antifilarial and antibacterial agents, have been proposed for these modified gold nanoparticles, however none of them has entered clinical trials yet. Both materials have their advantages and disadvantages, for instance, ceria particles are soluble at physiological pH, which is not always the case for materials coated with chitosan, that sometimes need an acid pH at higher concentrations. On the other hand, ceria materials can agglomerate or form crowns with proteins, compromising their bio-distribution, with is minimized in chitosan derived materials. To our knowledge, no apoptotic toxicity has been described for either of these gold nanoparticles. However, no coherent information can be found about their effect on cell viability and proliferation. While some works have found no impact in this regard, other studies have described a significant reduction in cell viability and proliferation for both materials [59,64–68]. The reason for these divergent results may be variation in the size, shape or net charge of nanoparticles, important features than have been well described to cause different effects on cells [69]. In this context, the development of new biomedical methodologies to monitor effects of nanoparticles, which can provide a better understanding of the mechanism related to certain therapeutic and/or toxic effects, may contribute to the optimized design of these materials. Specifically, in the case of functionalized gold nanoparticles, for which a wide range clinical applications have been proposed that are waiting to be transferred to clinics, a previous evaluation step of their effect on peripheral blood cells could provide a fast and effective filter before starting any clinical trial.

In our work, we first optimized a global protocol to analyze the metabolic profile of the three main types of blood cells (erythrocytes, polymorphonuclear leukocytes (PMNs) and mononuclear leukocytes (PBMCs)) that can be isolated in parallel from one patient sample. The cell fractions from different healthy volunteers (n = 4) were then separately exposed to nanoparticles based on gold, and stabilised on ceria nanoparticles or chitosan, with antioxidant properties. The metabolomic profile of the treated cells was thereupon compared with the profile of the untreated control cells. With this proof of concept, we intended to show that systematic metabolic changes can be detected in peripheral human blood cells after treatment with nanoparticles. This information can contribute, together with other toxicological studies or therapeutic data, to evaluate new nanomedicines in preclinical phases from a translational point of view, establishing a precedent in this field.

## Materials and methods

### Chemicals and materials

Solvents and reagents were purchased from: Sigma-Aldrich (Ficoll-Paque Plus, Ficoll-Paque Plus, PBS, fetal bovine serum, penicillin, streptomycin, amphotericin B, L-glutamine, chitosan, HAuCl_4_, sodium citrate, AgNO_3_, sodium phosphate dibasic dihydrate), Scharlab (methanol, chloroform, acetone, sodium hydroxide), Gibco (RPMI 1640 medium), Rhodia (CeO_2_), and Eurisotop (deuterated water, deuterated chloroform, deuterated trimethylsilyl propanoic acid, trimethylsilane). Materials were purchased from Scharlab, Life Technologies, and Falcon BD. Gases were supplied by Air-Liquide.

### Human subjects

The inclusion criteria were as follows: Caucasian healthy males (2) and females (2), 35–40 years, no alcoholics, no smoker, and no familiar with previous chronic diseases. All participants were recruited at the Outpatient’s Department of the Endocrinology Service at Vall d’Hebron University Hospital. The study was conducted according to the guidelines laid down in the Declaration of Helsinki, and all procedures were approved by the Ethics Committee of Vall d’Hebron University Hospital. Subjects have been properly instructed and have indicated that they consent to participate by signing the appropriate informed consent paperwork.

### Human peripheral blood cells isolation

The isolation of erythrocytes, PMNs and PBMCs leukocytes was carried out using a Ficoll-Paque gradient method [70]. 20 mL of peripheral blood freshly extracted from healthy volunteers was carefully poured into a tube with 40 mL of Ficoll and let stand for approximately 20 min, obtaining 3 phases. The upper ring consisted of leukocytes while erythrocytes concentrated as a pellet at the bottom. The leukocyte ring was carefully transferred in a tube with the same volume of Ficoll, avoiding mixing and, subsequently, centrifuged at 300 g for 25 min at 20°C. A pellet of PMNs cells and an intermediate ring with PBMCs cells were isolated separately through the following methodology. For the isolation of PBMCs, the PBMCs ring was transferred to a tube with the same volume of PBS and centrifuged at 300 g for 5 min at 20°C during 5 min. The supernatant was discarded and the pellet containing the PBMCs was kept on ice. For the isolation of PMNs, the pellet of PMNs, containing remainders of erythrocytes, was treated for 5 min with 1 mL of erythrocyte lysis buffer. Subsequently, the mixture was centrifuged at 300 g for 5 min at 20°C and the supernatant was discarded. The resulting pellet was resuspended in the same volume of PBS and centrifuged at 300 g for 5 min at 20°C. The supernatant was discarded and the pellet containing the PMNs was kept on ice. For the isolation of erythrocytes, the erythrocyte pellet was transferred to a tube with the same volume of PBS and centrifuged at 200 g for 5 min at 4°C without acceleration and brake. The supernatant was discarded and the erythrocyte pellet was washed with PBS again. Finally, after discarding the supernatant, the pellet containing the erythrocytes was kept on ice.

The resultant pellet of all the peripheral blood cells were: i) for direct metabolomic analysis, washed with PBS again and finally stored at -80°C after adding 0.5 mL of ice-cold methanol (for 20 million cells) or ii) for treatment with nanoparticles, resuspended in 1 mL of complete RPMI 1640 medium for cell counting and diluted in more RPMI 1640 until obtaining a solution of 5 million cells/mL (M/mL)

### Treatment of blood cells with nanoparticles

Twenty million PMNs and PBMCs, and 40 million erythrocytes were transferred to cell culture flasks at a concentration of 5 M/mL in a Telstar BIO Laminar flow cabinet. Cells were cultured in a RPMI 1640 medium consisting of 10% fetal bovine serum (FBS), 1% of antibiotic mixture (50 μg/mL Penicillin, 50 μg/mL Streptomycin), 1% (2.5 μg/mL) Amphotericin B and 2.05% L-Glutamine. To add the nanomaterials, the following procedures were carried out: a) AuCeO_2_: a 1 mg/mL dispersion of AuCeO_2_ in water was prepared, and then diluted to 20 μg/mL with medium. The pH was readjusted to 6.5 with 0.1 M of acetic acid. b) AuChi: a 1 mg/mL solution of AuChi in 0.1% acetic acid was prepared, and then diluted to 20 μg/mL with medium. The pH was readjusted to 6.5 with 0.1 M of NaOH. For control samples, the pH of the medium was directly adjusted to 6.5 with 0.1 M of acetic acid. Flasks were then incubated in an IGO 150 (Jouan, Saint-Herblain, France) incubator during 24 h without stirring at 37°C and 5% of CO_2_. After incubation with the different treatments, the content of each flask of cells was transferred to a falcon tube and the remaining cells were scraped off in PBS and added to the tube. Leukocyte cells were centrifuged at 300 g and 20°C during 5 min and erythrocytes at 200 g and 4°C during 10 min without acceleration and brake. Supernatants were then discarded, the pellets resuspended in the same volume of PBS and centrifuged again under the same conditions. Then, leukocytes cells were centrifuged at 11000 g and 20°C during 5 min and erythrocytes at 200 g and 4°C during 10 min. Supernatants were discarded, 0.5 mL of ice-cold methanol was added to the pellets, that were stored at -80°C until performing the extraction for ^1^H-NMR analysis.

### Extraction of polar and nonpolar metabolites for ^1^H-NMR experiments

Frozen samples were placed on ice, allowed to that for 5 min, and then subjected to an extraction procedure. Then, 250 μl of chloroform at 4°C per 20 million cells were added to the corresponding pellets and let stand for 30 min. Samples were then homogenized with a vortex, cells resuspended with a pipette and transferred to a 1.5 ml tube. For uniform cell breakage, samples were submitted to three freeze-thaw cycles with liquid nitrogen. Then, 400 μL of distilled water and 400 μL of chloroform were added to each sample which then was vortexed. Samples were then centrifuged at 13000 g for 20 min at 4°C to separate phases. The solution was separated into an upper water/methanol phase (with polar metabolites, aqueous phase), an interphase containing mainly proteins, DNA/RNA and cell membranes, and a lower chloroform/methanol phase (with lipophilic compounds, organic phase). To obtain dry extracts, the aqueous phase was lyophilized overnight and the organic phase removed using a speed vacuum concentrator. Extracts were stored at -80°C until sample preparation for the ^1^H-NMR experiments.

### ^1^H-NMR experiments

Frozen cell pellets were placed on ice and allowed to thaw for 5 min. To the aqueous phase was solubilised in 550 μL of phosphate buffer (100 mM Na_2_HPO_4_ pH 7.4, in D_2_O) containing 0.1 mM of deuterated trimethylsilyl propanoic acid (TSP-D4). The organic extract was dissolved in 550 μL of cold deuterated chloroform (CDCl_3_ with 0.03% trimethylsilyl propanoic acid, TMS). Samples were stored at 4°C, equilibrated at RT for 15 min before analysis and analysed the same day. ^1^H-NMR spectra of extracts were recorded at 27°C on a Bruker AVII600 MHz spectrometer using a 5 mm TCI cryoprobe and processed using Topspin3.2 software (Bruker GmbH, Karlsruhe, Germany). ^1^H 1D noesy NMR spectra were acquired with 256 free induction decays (FIDs), 64k data points, a spectral width of 30 ppm and a relaxation delay of 4s. Water presaturation was applied for aqueous samples. The FID values were multiplied by an exponential function with a 0.5 Hz line broadening factor. Total Correlation Spectroscopy (TOCSY) and multiplicity Heteronuclear Single Quantum Correlation (HSQC) were performed on representative samples with 256–512 t1 increments, 32–96 transients and a relaxation delay of 1.5 s. TOCSY spectra were recorded using a standard MLEV-17 pulse sequence with mixing times (spin-lock) of 65 ms.

### Synthesis and characterization of gold-chitosan nanoparticles (AuChi)

AuChi nanoparticles were synthesized as previously reported [60]. Briefly, 200 mg of low molecular weight chitosan were added to 100 mL of miliQ water containing 1% of acetic acid. Then, the mixture was heated at 90°C with vigorous stirring until complete dissolution of chitosan. Subsequently, 1.3 mL of a 9.6 mM HAuCl_4_ x 3H_2_O aqueous solution was added slowly to the chitosan solution and the mixture was stirred for 5 min. Afterward, 250 μL of a 0.1 M sodium citrate solution was added and the mixture was stirred for another 5 min. Later, the mixture was quickly cooled in a water-ice bath. Then, the solution was filtered through a 0.22 μm cellulose filter and characterized. The hydrodynamic size and the zeta potential were determined by dynamic light scattering (DLS) (Zetasizer Nano ZS (Malvern Instrument, UK)). The gold nanoparticle size was determined by high resolution transmission electron microscopy (HR-TEM) (Philips CM300FEG 100 kV). Finally, the content of gold was determined by inductively coupled plasma (ICP) (Varian 715-ES ICP-Plasma).

### Synthesis and characterization of gold-ceria nanoparticles (AuCeO_2_)

AuCeO_2_ nanoparticles were synthesized following a protocol previously described by our group [59]. Briefly, 200 mg of HAuCl_4_ x 3H_2_O were dissolved in 40 mL of distilled water at RT. Then, a solution of 0.2 M NaOH was added until pH = 10 while the mixture was stirred vigorously. Once the pH was stable, a suspension of 1.0 g CeO_2_ in 13 mL of distilled water was added slowly, adjusting the pH of the mixture to 10 with 0.2 M NaOH. When the pH was stable, the mixture was stirred vigorously overnight. After that, the dispersion was filtered and washed with several litres of distilled water until no traces of chlorides were detected by the AgNO_3_ test. Then, the solid was washed with 2x100 mL of acetone, dried and placed in a furnace. The sample was then heated from RT to 300°C at a rate of 8°C/min during 4.5 h, in presence of H_2_. After that, the furnace was shut down until reaching room temperature. Then, nanoparticles were dissolved on PBS and the resultant solution was filtered through a 0.22 μm cellulose filter. The gold nanoparticle size was determined by HR-TEM (Philips CM300FEG 100 kV). The hydrodynamic size and zeta potential of the solid were analyzed by DLS (Zetasizer Nano ZS (Malvern Instrument, UK)). Finally, the content of gold was determined by ICP (Varian 715-ES ICP-Plasma).

### Data analysis and statistics

^1^H-NMR spectra were transformed with a 0.5 line-broadening, and manually baseline and phase corrected with Topspin 3.2. NMR signals of TSP-D_4_ (polar spectra) and TMS (non-polar spectra) were referenced to 0 ppm. For metabolite identification, the ^1^H and ^13^C chemical shift values and multiplicity of the signals were compared with reference data from the spectral databases Human Metabolome Database and the Biological Magnetic Resonance Bank and several literature reports [29,71,72]. The assignment NAD, NADH, NADP, NADPH, ATP, ADP, acetoacetate and sarcosine, was confirmed by spiking the sample with reference compounds. Spectra were normalized to total intensity to minimize the differences in concentration and experimental error during the extraction process. Optimal integration regions were defined for each metabolite, trying to select signals without overlapping. Integration was performed with MestreNova 8.1 utilization the GSD deconvolution option. In the study with gold-nanoparticle treatments, p-values were calculated with the non-parametric Mann-Whitney U test with IBN SPSS statistics 21. Pathway analysis was performed with Metaboanalyst [73].

## Results and discussion

### Optimisation of blood cell isolation, treatment and metabolite extraction

A protocol for the isolation of the different human blood cell types and their metabolites was initially optimized. To this end, peripheral blood was extracted from healthy individuals and the three mayor blood cell fractions (RBCs, PMNs and PBMCs) were separated. Two different methods were tested for evaluating the separation of the different fractions: the Ficoll-Paque gradient method, and dextran followed by the Ficoll-Paque gradient method. No significant differences in the quality of the metabolomic profile obtained using both procedures were found. Therefore, the Ficoll-Paque gradient method was selected for its simplicity and minimal sample handling. Another important factor to consider is that erythrocytes are particularly fragile, so they must be centrifuged at a lower speed without acceleration or braking.

**Fig 1:**
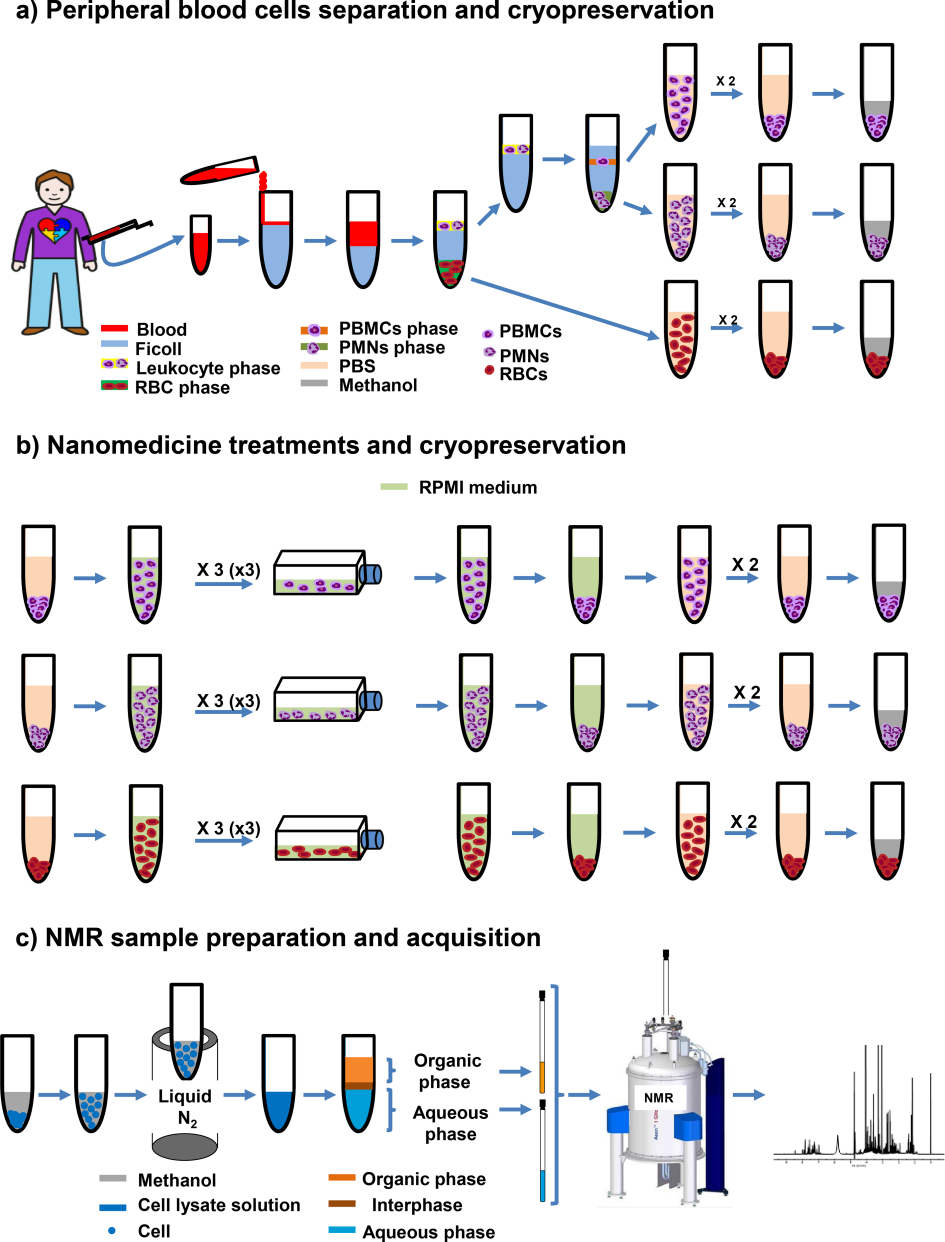
Strategy for peripheral blood cell isolation (PBMCs, PMNs and erythrocytes), nanoparticle treatment, polar and no-polar metabolite extraction, and ^1^H-NMR analysis. PBMCs, PMNs and erythrocytes samples were isolated from peripheral blood of healthy human individuals. Samples of each cell type were split an aliquot (20 million cells) for characterization (a), and another aliquot (40 million cells) for nanoparticle treatments (b). Finally, polar and nonpolar were extracted and the ^1^H-NMR metabolic profiles determined (c).

After achieving phase separation, cells were submitted to treatment with nanoparticles (see details in the Supplementary Information). Three different concentrations of cells were tested: 30, 15 and 5 M/mL. Agglomeration and cell death was observed at the two higher concentrations (30 and 15 M/mL), but not at the lowest concentration (5 M/mL). Finally, cells were harvested and subjected to an extraction process with methanol, chloroform and water, a method that had been successfully applied in previous metabolomics studies [74]. This process is extremely sensitive to the elimination of metabolites from the medium that could interfere with the analysis, a process that was achieved by washing with PBS. Furthermore, cold methanol (-20°C) was added to effectively quench the metabolism of cells. Solvent amounts were optimised to 500 μL methanol (added directly after cell harvesting), 650 μL of chloroform (added in two times, before and after cell breakage) and 400 μL of water (added after cell breakage, to avoid ice-building during the freeze-thaw cycles) per 20 million cells. Smaller solvent volumes produced poorer extractions yields and a less efficient phase separation, while bigger volumes showed no improvement. As a result of the extraction, two different, aqueous and organic, fractions were obtained with polar and non-polar metabolites, respectively. For the NMR analysis, cell extracts were dried and later on dissolved in deuterated solvents to improve spectrum quality, which contained internal standards (for quantification), and were buffered in the aqueous phase (for pH control). The whole process is summarised in Fig 1.

### Metabolic profile of RBCs, PMNs, and PBMCs blood cells

**Fig 2:**
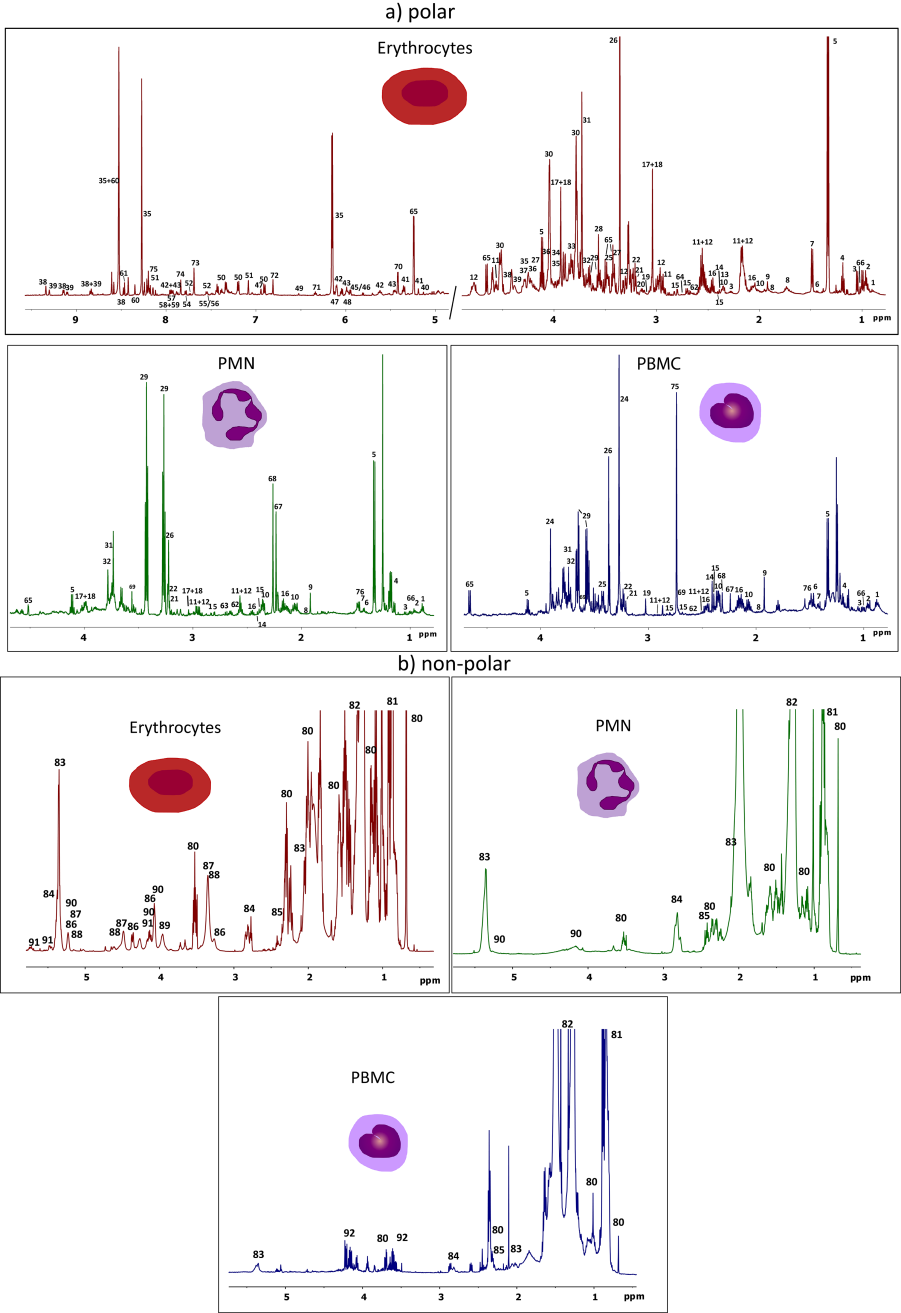
^1^H-NMR spectra of erythrocytes, PMNs and PBMCs. Polar (a) and non polar (b) ^1^H-NMR metabolomic profiles of extracts of the main types of peripheral blood cells. Metabolite assignments are indicated with the following numbers: 1) 2-hydroxybutyrate, 2) leucine, 3) valine, 4) ethanol, 5) lactate, 6) 2-aminoisobutyrate, 7) alanine, 8) lysine, 9) acetate, 10) glutamate, 11) reduced glutathione (GSH), 12) oxidized glutathione (GSSG), 13) pyroglutamate, 14) pyruvate, 15) succinate, 16) glutamine, 17) creatine, 18) phosphocreatine, 19) malonate, 20) spermidine/spermine, 21) phosphocholine (PC), 22) glycerophosphocholine (GPC), 23) carnitine, 24) betaine, 25) taurine, 26) methanol, 27) proline, 28) glycine, 29) glycerol, 30) ascorbate, 31) guanidino/guanido acetate, 32) 6-phosphogluconate 33) glycolate, 34) phosphoethanolamine, 35) ATP, 37) AMP, 38) NAD+, 39) NADP+, 40) trehalose, 41) phosphoenolpyruvate, 42) UDP-glucose, 43) UDP-NAG, 43) NADH, 44) uracil/tryptophane, 45) GDP, 46) GTP, 47) NADPH, 48) CTP, 49) fumarate, 50) tyrosine, 51) histidine/histamine, 52) tryptophane, 53) phenylalanine, 54) guanosine, 55) xanthine, 56) guanine, 57) hypoxanthine, 58) CTP, 59) CDP, 60) ADP, 61) formate, 62) methionine, 63) aspartate, 64) malate, 65) glucose, 66) isoleucine, 67) acetoacetate, 68) methylacetoacetate, 69) sarcosine, 71) thymidine, 72) hydroquinone, 73) pyridoxamine, 74) 4-pyridoxate, 75) nicotinamide, 76) N-Methyl-a-aminoisobutyric acid, 80) cholesterol, 81) lipid CH_3_-, 82) lipid -CH_2_-, 83) fatty ester -CH_2_CH_2_COO-, 84) polyinsaturated fatty acids (PUFA), 85) monoinsaturated fatty acids (MUFA), 86) phosphatidylethanolamine, 87) phosphatidycholine, 88) acylglycerophosphoserine, 89) phospholipids, 90) TAG, 91) spingosine, 92) fatty ester—CH_2_OCO-.

^1^H-NMR spectra corresponding to the aqueous and organic extracts of the three peripheral blood cell types are shown in Fig 2. A detailed assignment of the different spectra was performed based on the 1D and 2D-NMR spectra acquired in this study, as well as the general information available from public databases, since no exhaustive information regarding the metabolic content of these blood cells could be found in the literature. Thus, it was possible to identify more than 80 different polar and non-polar metabolites, or functional groups (Fig 2). The main metabolites identified in the aqueous phase were amino acids, sugars, organic acids and nucleotides, whereas in the organic fraction it was possible to identify different types of lipids, such as mono and polyunsaturated lipids, di- and triglycerols, phospholipids, as well as cholesterol. This is the first time that the metabolic profile of different kind of blood cells has been systematically determined by NMR, and the results are coherent with data obtained in previous studies using GC-MS and LC-MS [75,76].

It should be stressed that each cell type had a specific metabolomic profile. RBCs, for instance, exhibited large amounts of lactate, glucose, glutathione, ATP/ADP, ascorbic acid, creatine and phospholipids. The presence of several of these metabolites has been previously described in RBCs by other techniques, and their level alterations are associated with diseases such as sickle cell, Alzheimer’s disease, Wilson’s disease or anaemia [77–81]. Both PMNs and PBMCs leukocytes contained very large amounts of glycerol, a compound that, among other functions, is a vital osmoprotective agent for cells in suspension. The levels of metabolites such as glucose, pyruvate and succinate were higher in PBMCs than in PMNs, perhaps reflecting the importance of oxidative bioenergetic metabolism of lymphocytes (main fraction of PBMCs) under basal conditions [82]. Moreover, it has been demonstrated that the bioenergetic metabolism of neutrophils (main fraction of PMNs) is mainly glycolytic [82]. Accordingly, lactate concentrations were significantly higher in this blood cell type.

In summary, metabolomics profiles obtained by ^1^H-NMR provide relevant information of the specific metabolism associated with each cell type, that could be extremely useful from a clinical point of view.

### Effect of nanoparticle treatment on the metabolic profile of RBCs, PMNs, and PBMCs

**Fig 3:**
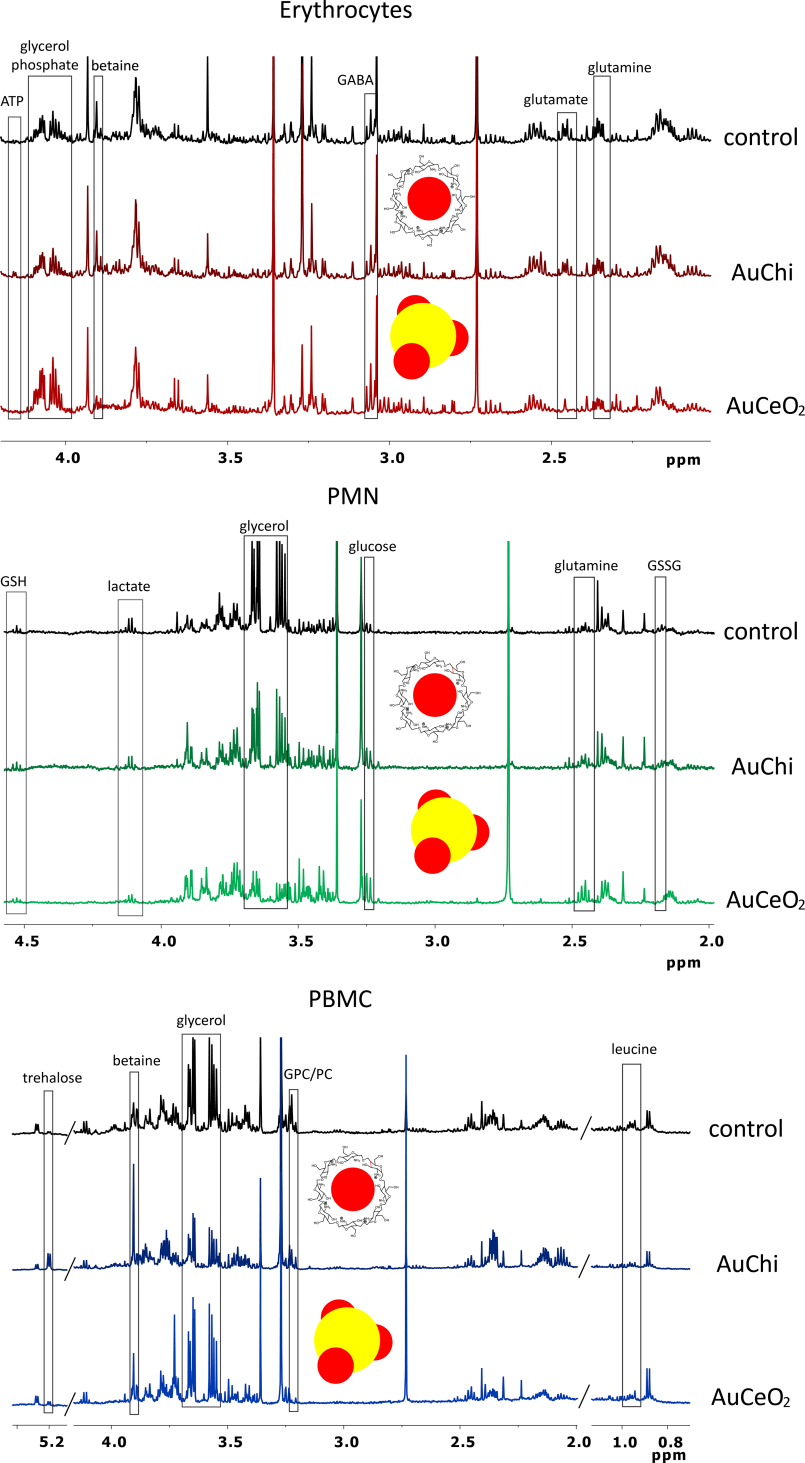
^1^H NMR spectra of the aqueous extracts of RBCs, PMNs and PBMCs after treatment with nanoparticles. Changes observed in the metabolomic profile of the main peripheral blood cells after 3h of treatment with vehicle, AuChi and AuCeO_2_ nanoparticles.

Once a reliable protocol for evaluating the metabolomic profile of each cell type was established, we evaluated its possible application in nanomedicine. To this end, the effects caused by chitosan-capped gold nanoparticles (AuChi) and gold nanoparticles stabilised on ceria nanoparticles (AuCeO_2_), two promising type of gold nanoparticles for biomedicine, were assessed. A detailed physical characterization of both nanoparticles revealed mean sizes, determined by HR-TEM, of 5.65 nm (AuChi) and 5.90 nm (AuCeO_2_) (S1 File, Fig A and D, respectively). Moreover, zeta potentials of + 17 mV (AuChi) and -19 mV (AuCeO_2_), as well as hydrodynamic sizes of 205 nm (AuChi) and 133 nm (AuCeO_2_), that are optimal values for biomedical applications, were determined by DLS (S1 File, Fig B and C, respectively). Finally, the gold content of both nanoparticles, characterized by ICP, was 1.2% (AuChi) and 0.8 (AuCeO_2_). Pictorial representations of the two-particle systems can be found in Fig 3. The non-toxicity of the nanomaterials was confirmed with an MTT-assay (S2 File).

A pilot study was then conducted to evaluate the impact of both nanoparticles in each cell type. Thus, blood was extracted from healthy volunteers and subjected to the previously described protocol (Fig 1). To evaluate the effect that the coating had on the blood cells, control experiments with commercial chitosan and CeO_2_ were also carried out. The representative ^1^H-NMR spectra of RBCs, PMNs and PBMCs from freshly peripheral human blood after being exposed for 24 h to AuChi, AuCeO_2_ and the vehicle are shown in Fig 3. Significant differences in the metabolomic profiles of blood cells after treatment with both nanomaterials were detected, as shown in Tables 1–3 and Tables A–C in S3 File. To get an overview of the affected pathways, pathway analysis was performed with the program metaboanalyst, using metabolites that change significantly as input data (Fig A to F in S4 File). In general, it was found that the metabolic changes induced by AuChi were less pronounced than those associated with AuCeO_2_. This result is in agreement with previous studies indicating a lower AuChi toxicity compared with AuCeO_2_ [59,67,68]. Interestingly, it was also found that the metabolic impact of the nanoparticles was cell-type specific, an indication of the existence of different mechanism of actions of the nanoparticles in each blood cell type.

**Table 1:** Significant changes in RBCs.

Metabolite	AuChi treatment	AuCeO_2_ treatment
ATP	↑	↑
betaine	-	↓
GABA	-	↑
glutamate	-	↓
glutamine	-	↓
glycerol phosphate	-	↑
glucose	↑	-
cholesterol	-	↑
PUFA	-	↑

Significant changes (↑: metabolite increased vs control; ↓: metabolite decreased vs control) (↑ or ↓: p < 0.05 > 0.01) in the metabolic profile of RBCs after treatment with AuChi and AuCeO_2_ nanoparticles.

**Table 2:** Significant changes in PMNs.

Metabolite	AuChi treatment	AuCeO_2_treatment
betaine	↑	↑
lactate	↓	↓
glutamine	↑	↑↑
GSSG	↓	↓
glucose	-	↑
glycerol	↓	↓↓
GSH	↑	-
lipid CH_2_	-	↓

Significant changes (↑: metabolite increased vs control; ↓: metabolite decreased vs control) (↑ or ↓: p < 0.05 > 0.01; ↑↑ or ↓↓: p < 0.01) in the metabolic profile of PMNs after treatment with AuChi and AuCeO_2_ nanoparticles.

**Table 3:** Significant changes in PBMCs.

Metabolite	AuChi treatment	AuCeO_2_ treatment
formate	↓	-
trehalose	↑	-
betaine	↑	-
PC	↓	↓↓
GPC	-	↓
leucine/valine/isoleucine	↓	↑
glycerol	↓	-
cholesterol	-	↓
glycerides	↓	-
MUFA	-	↓
PUFA	-	↓

Significant changes (↑: metabolite increased vs control; ↓: metabolite decreased vs control) (↑ or ↓: p < 0.05 > 0.01; ↓↓: p < 0.01) in the metabolic profile of PBMCs after treatment with AuChi and AuCeO_2_ nanoparticles.

The antioxidant effect of nanoparticles, as indicated by an increase in the GSH/GSSG ratio after treatment with AuChi nanoparticles, was better reflected in PMN cells as they contain a large number of mitochondria as other cell types. Simultaneously, a drop in lactate was observed, probably reflecting a reduced glucose conversion (main energy source in neutrophils) into lactate, in parallel with an increased insertion of pyruvate into the oxidative citric acid cycle. These cells have been reported to use glycerol-phosphate for energy production in mitochondria [83]. Thus, antioxidant materials could have activated glycerol-phosphate oxidation in mitochondria, a process that would explain the observed reduction of glycerol levels. In this situation, the observed increase of betaine levels, a known osmolyte, could compensate the decrease of gycerol levels [84]. Another important observation was the decrease in glutamine levels, revealing an alteration in glutaminolysis, a process that occurs at high rates in immune system cells [83].

In general, the antioxidant impact of the nanoparticles was less pronounced in PBMC cells than in PMN cells, an indication perhaps of the fact that lymphocytes already rely on an oxidative metabolism for energy production. Also in this case, a drop in glycerol and an increase in betaine upon AuChi treatment were observed, but these effects did not take place for AuCeO_2_. The decrease in phosphocholine derivatives, glycerides (AuChi) and MUFA, PUFA and cholesterol (AuCeO_2_) could also be indicative for an alteration in the glycerophospholipid metabolism of PBMCs (Table E and F in S4 File).

Metabolic changes induced by nanoparticles on erythrocytes were less pronounced, a process that could be explained by the fact that these cells do not possess organelles, (e.g., mitochondria), the main target of the antioxidant properties of the gold nanoparticles. RBCs already possess a strong antioxidant system to protect the Hemo group, so minor antioxidant effects cannot be easily detected. Therefore, erythrocytes may be optimal sensors for a strong antioxidant or oxidative effect, but this was not the case for either material employed herein. Nevertheless, higher ATP levels were detected upon both treatments, a process that could reflect an altered energetic metabolism of the cells induced by the gold nanoparticles. Also amino acid metabolism seemed to be affected after treatment with AuCeO_2_, while AuChi seems to interfere with sugar metabolism (Table A and B in S4 File).

Several of the detected changes, especially those observed for AuCeO_2_, have also been detected in cultured cells treated with different kind of nanoparticles, including the increase in different amino acids (valine, leucine, isoleucine, glutamate or glutamine) and the decrease in PC, GPC, formate and ATP [85–88]. The increase of the GSH/GSSG seems to be specific for nanoparticles with anti-oxidant properties, such as gold and silver nanoparticles, as it has not been detected for copper, cobalt or titanium nanoparticles.

Control experiments with CeO_2_ and chitosan revealed that the observed effects were mainly due to the combination of Au with chitosan and CeO_2_, as very few significant metabolic changes were observed after treatment with chitosan and CeO_2_ in the absence of Au (Tables D–F in S3 File). In the case of RBCs, ATP also increased, which could mean that part of the alteration in the energetic metabolism of these cells can be already be induced by the support/coating materials. They also seem to cause a slight decrease in the GSH/GSSG ratio in these cells, which seems to be compensated by the antioxidant effect of Au when the materials are combined with gold. For PMNs, a tendency for glucose and GSH to increase, and lactate to decrease seem to be present when they are treated with chitosan and CeO_2_, but these changes are only significant when Au is introduced. Changes in PBMCs (lactate and acetoacetate) seem not to be related with the changes that take place in the presence of gold nanoparticles.

In summary, results indicate that chitosan gold nanoparticles possess a relevant oxidative capacity, especially on PMNs cells, while this effect was much less pronounced for AuCeO_2_. Furthermore, although both nanoparticles have been described as biocompatible, they have an impact on several metabolic routes (e.g., the bioenergetic metabolism of cells, amino acid metabolism, sugar metabolism). Therefore, these characteristics should be taken into consideration for the clinical development of these materials.

## Conclusions

This work presents a preliminary study aiming to contribute to the design and evaluation of nanoparticles for biomedical applications. A protocol was developed for the monitoring of a large number of polar and non-polar metabolites from three different types of peripheral blood cells (RBCs, PMNs, and PBMCs). The protocol was successfully applied to the evaluation of the effect of nanomaterials on these cells, an application that could have important implications from a traslational point of view. Treatment of the three blood cell types with gold nanoparticles, which were stabilized on ceria or chitosan, revealed different systematic changes in the metabolic profile of the cells, which were related to the antioxidant effect of the materials but also reflected alterations in other metabolomics pathways. In combination with other analytical and biochemical techniques, this tool could provide relevant information about the mechanism of action and side effects of new nanomedicines, which in turn could offer a better way for the optimization and evaluation of these nanoparticles. Interesting further projects include the analysis of the effect of different types of nanoparticles, especially those that are already in clinical trials, and the comparison of the effect that they have on the blood from patients and from healthy individuals.

## Supporting information

S1 File Characterization of AuChi and AuCeO2 nanoparticles. (DOCX)

S2 File Cellular viability study with the MTT assay. (DOCX)

S3 File Data tables of metabolomics changes. (DOCX)

S4 File Pathway analysis. (DOCX)
