# Correction: Assessment of gold nanoparticles on human peripheral blood cells by metabolic profiling with ^1^H-NMR spectroscopy, a novel translational approach on a patient-specific basis

**DOI:** 10.1371/journal.pone.0189748

**Published:** 2017-12-11

**Authors:** Martina Palomino-Schätzlein, Hermenegildo García, Patricia Gutiérrez-Carcedo, Antonio Pineda-Lucena, José Raul Herance

There is an error in affiliation 3 for authors Patricia Gutiérrez-Carcedo and José Raul Herance. Affiliation 3 should be:

Grup de Recerca en Imatge Mèdica Molecular, Diabetes and Metabolism Vall d’Hebron Research Institute, CIBERDEM, CIBBIM-Nanomedicine, Departament de Medicina, Universitat Autònoma de Barcelona, Barcelona, Spain.
