# Supplementary material for: Assessment of gold nanoparticles on human peripheral blood cells by metabolic profiling with 1H-NMR spectroscopy, a novel translational approach on a patient-specific basis
Source: PLoS One. 2017 Aug 9;12(8):e0182985. doi: 10.1371/journal.pone.0182985 (PMC5549967; doi:10.1371/journal.pone.0182985)
Supplement: S1 File — (DOCX) [file pone.0182985.s001.docx]

Supporting Information – S1 File

**Characterization of AuChi and AuCeO_2_ nanoparticles**

Transmission electron microscopy (TEM) images were acquired by a Philips CM300FEG system with an operating voltage of 100 kV by placing microdrops of nanoparticles solution directly onto a copper grid coated with carbon film (200 mesh). Dynamic Light Scattering used to determine the size and zeta potential of colloidal aqueous solutions of nanoparticles was performed on a Zetasizer Nano ZS (Malvern Instrument, UK). 5 mg of nanoparticles were suspended in 100 mL for measurements, which were carried out in an automatic mode. The values of size and zeta potential are the average of 30 runs. The centrifuge used was a Hettich-Zentrifugen one (Tuttlingen, Germany). A 30 mg sample for Inductively Coupled Plasma (ICP) analysis was dissolved in a mixed acid solution 1mL HF (40%), 1 mL HNO_3_ (65%), 3mL HCl (30%) at room temperature 24h and then diluted to 60 mL with distilled water and sent to a Varian 715-ES ICP-Plasma.

**AuChi**


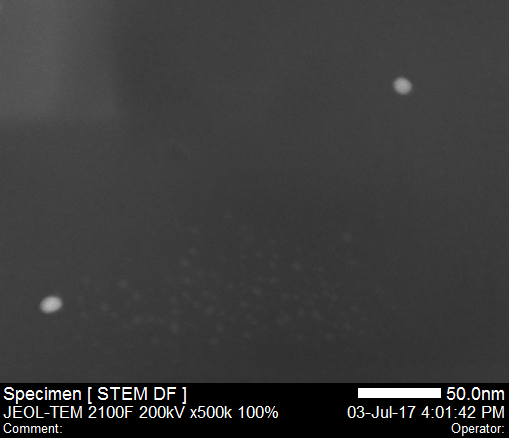


**S1 Fig A. High resolution transmission electron microscopy recorded for AuChi.** The average size of gold nanoparticle coated with chitosan was determined after 100 measures of nanoparticles.


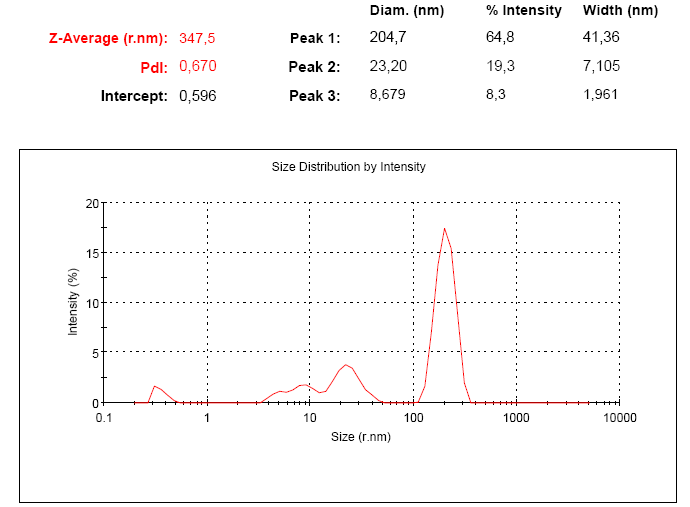


**S1 Fig B. Dynamic light scattering recorded for AuChi.** This analysis to determine the hydrodynamic size of AuChi was performed using a 20 µg/mL solution in PBS.

**AuCeO_2_**


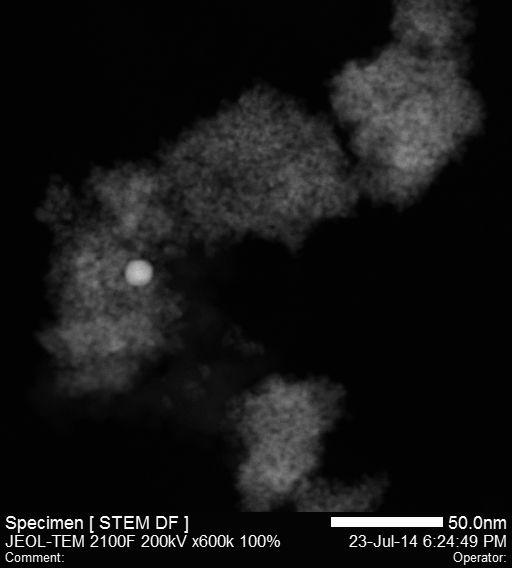


**S1 Fig C. High resolution transmission electron microscopy recorded for AuCeO_2_.** The average size of gold nanoparticle supported on ceria nanoparticles was determined after 100 measures of nanoparticles.


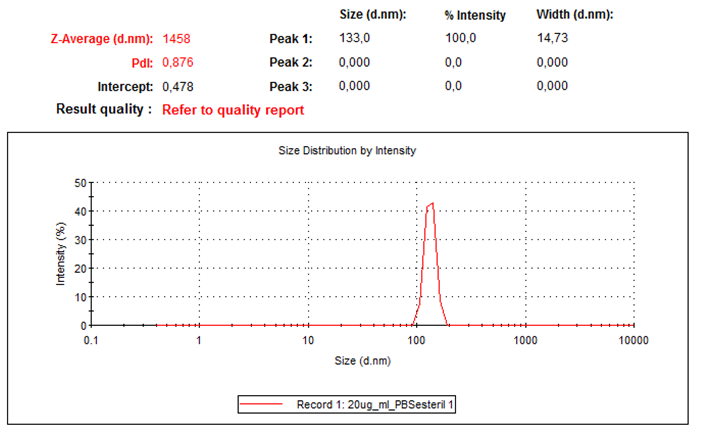


**S1 Fig D. Dynamic light scattering recorded for AuCeO_2_.** This analysis was performed using a 20 µg/mL solution of AuCeO_2_ in PBS to determine the hydrodynamic size of these nanoparticles.
