# Supplementary material for: Assessment of gold nanoparticles on human peripheral blood cells by metabolic profiling with 1H-NMR spectroscopy, a novel translational approach on a patient-specific basis
Source: PLoS One. 2017 Aug 9;12(8):e0182985. doi: 10.1371/journal.pone.0182985 (PMC5549967; doi:10.1371/journal.pone.0182985)
Supplement: S2 File — (DOCX) [file pone.0182985.s002.docx]

Supporting Information – S2 File

**Cellular viability study with the MTT assay**

HeLa cells were harvested with 200 µl of total volume in 96-well plates at 40000 cells/well at 24 h and 80 % of confluence. Incubation in the presence of CeO_2_, AuCeO_2_, Chi and AuChi was done for 24 h, after which the medium was refreshed and 10 μl/well of 3-[4,5-dimethylthiazol-2-yl]-2,5-diphenyl tetrazolium bromide was added at 37 ºC for 4 h. After that, 100 μL/well of solubilization solution was poured into wells containing cells and was incubated overnight at 37 °C. Then, absorbance was detected using a plate-reader spectrophotometer. Results were obtained by subtracting the intensity of the signal at 690 nm from the 570 nm absorbance.

**S2 Fig. MTT assay.** Absorbance detected from HeLa cells after incubation with different nanomaterials during 24h.
