# Supplementary material for: Assessment of gold nanoparticles on human peripheral blood cells by metabolic profiling with 1H-NMR spectroscopy, a novel translational approach on a patient-specific basis
Source: PLoS One. 2017 Aug 9;12(8):e0182985. doi: 10.1371/journal.pone.0182985 (PMC5549967; doi:10.1371/journal.pone.0182985)
Supplement: S3 File — (DOCX) [file pone.0182985.s003.docx]

Supporting Information – S3 File

**Data tables of metabolomic changes**

**S3 Table A. Significant metabolites for RBCs.** Normalized concentration values of metabolites changing significantly in RBCs after treatment with AuChi and AuCeO_2_. p-values were obtained applying the Mann-Whitney U test (n = 6). CT = control, SD = standard deviation.

**S3 Table B. Significant metabolites for PMNs.** Normalized concentration values of metabolites changing significantly in PMNs after treatment with AuChi and AuCeO_2_. p-values were obtained applying the Mann-Whitney U test (n = 6). CT = control, SD = standard deviation.

**S3 Table C. Significant metabolites for PBMCs.** Normalized concentration values of metabolites changing significantly in PBMCs after treatment with AuChi and AuCeO_2_. p-values were obtained applying the Mann-Whitney U test (n = 6). CT = control, SD = standard deviation.

**S3 Table D. Significant metabolites for RBCs.** Normalized concentration values of metabolites changing significantly in RBCs after treatment with chitosan and ceria. p-values were obtained applying the Mann-Whitney U test (n = 6). CT = control, SD = standard deviation

**S3 Table E. Significant metabolites for PMNs.** Normalized concentration values of metabolites changing significantly in PMNs after treatment with chitosan and ceria. p-values were obtained applying the Mann-Whitney U test (n = 6). CT = control, SD = standard deviation

**S3 Table F. Significant metabolites for PBMCs.** Normalized concentration values of metabolites changing significantly in PBMCs after treatment with chitosan and ceria. p-values were obtained applying the Mann-Whitney U test (n = 6). CT = control, SD = standard deviation
