# Supplementary material for: Assessment of gold nanoparticles on human peripheral blood cells by metabolic profiling with 1H-NMR spectroscopy, a novel translational approach on a patient-specific basis
Source: PLoS One. 2017 Aug 9;12(8):e0182985. doi: 10.1371/journal.pone.0182985 (PMC5549967; doi:10.1371/journal.pone.0182985)
Supplement: S4 File — (DOCX) [file pone.0182985.s004.docx]

Supporting Information – S4 File

**Pathway analysis**

**S4 Table A. Pathway analysis of RBCs after AuChi treatment.** Summary of altered metabolic pathways in RBCs cells after treatment with AuChi nanoparticles, obtained with the pathways analysis tool of metaboanalyst. Pathways with are significantly altered are labelled in red.

**S4 Table B. Pathway analysis of RBCs after AuCeO_2_ treatment.** Summary of altered metabolic pathways in RBCs cells after treatment with AuCeO_2_ nanoparticles, obtained with the pathways analysis tool of metaboanalyst. Pathways with are significantly altered are labelled in red.

**S4 Table C. Pathway analysis of PMNs after AuChi treatment.** Summary of altered metabolic pathways in PMNs cells after treatment with AuChi nanoparticles, obtained with the pathways analysis tool of metaboanalyst. Pathways with are significantly altered are labelled in red.

**S4 Table D. Pathway analysis of PMNs after** **AuCeO_2_ treatment.** Summary of altered metabolic pathways in PMNs cells after treatment with AuCeO_2_ nanoparticles, obtained with the pathways analysis tool of metaboanalyst. Pathways with are significantly altered are labelled in red.

**S4 Table E. Pathway analysis of PBMCs after AuChi treatment.** Summary of altered metabolic pathways in PBMCs cells after treatment with AuChi nanoparticles, obtained with the pathways analysis tool of metaboanalyst. Pathways with are significantly altered are labelled in red.

**S4 Table F. Pathway analysis of PBMCs after AuCeO_2_ treatment.** Summary of altered metabolic pathways in PBMCs cells after treatment with AuCeO_2_ nanoparticles, obtained with the pathways analysis tool of metaboanalyst. Pathways with are significantly altered are labelled in red.
